# Beta-Amyloid Peptides Enhance the Proliferative Response of Activated CD4^+^CD28^+^ Lymphocytes from Alzheimer Disease Patients and from Healthy Elderly

**DOI:** 10.1371/journal.pone.0033276

**Published:** 2012-03-12

**Authors:** Agnieszka Jóźwik, Jerzy Landowski, Leszek Bidzan, Tamas Fülop, Ewa Bryl, Jacek M. Witkowski

**Affiliations:** 1 Department of Pathophysiology, Medical University of Gdańsk, Gdańsk, Poland; 2 Department of Psychiatry and Neurotic Disorders, Medical University of Gdańsk, Gdańsk, Poland; 3 Department of Developmental Psychiatry, Psychotic Disorders, and Geriatric Psychiatry, Medical University of Gdańsk, Gdańsk, Poland; 4 Immunology Program, Geriatric Division, Faculty of Medicine, Research Center on Aging, University of Sherbrooke, Sherbrooke, Quebec, Canada; University of North Dakota, United States of America

## Abstract

Alzheimer's disease (AD) is the most frequent form of dementia among elderly. Despite the vast amount of literature on non-specific immune mechanisms in AD there is still little information about the potential antigen-specific immune response in this pathology. It is known that early stages of AD include β-amyloid (Aβ)- reactive antibodies production and inflammatory response. Despite some evidence gathered proving cellular immune response background in AD pathology, the specific reactions of CD4^+^ and CD8^+^ cells remain unknown as the previous investigations yielded conflicting results. Here we investigated the CD4^+^CD28^+^ population of human peripheral blood T cells and showed that soluble β-amyloids alone were unable to stimulate these cells to proliferate significantly, resulting only in minor, probably antigen-specific, proliferative response. On the other hand, the exposure of *in vitro* pre-stimulated lymphocytes to soluble Aβ peptides significantly enhanced the proliferative response of these cells which had also lead to increased levels of TNF, IL-10 and IL-6. We also proved that Aβ peptide-enhanced proliferative response of CD4^+^CD28^+^ cells is autonomous and independent from disease status while being associated with the initial, *ex vivo* activation status of the CD4^+^ cells. In conclusion, we suggest that the effect of Aβ peptides on the immune system of AD patients does not depend on the specific reactivity to Aβ epitope(s), but is rather a consequence of an unspecific modulation of the cell cycle dynamics and cytokine production by T cells, occurring simultaneously in a huge proportion of Aβ peptide-exposed T lymphocytes and affecting the immune system performance.

## Introduction

Although it is clear that Alzheimer's disease (AD) is a degenerative brain disorder, the role of immune system in the disease pathogenesis is indisputable (reviewed e.g. in [1]). AD leads to irreversible cognitive decline manifesting mainly in memory impairment, and lately by behavioral changes. Typical changes in the brain include extracellular deposition of fibrillar 42-aa form of β-amyloid (Aβ_1–42_) surrounded by dystrophic neurites forming senile plaques. Inflammation has a direct contribution to neurodegeneration associated with AD as shown by numerous epidemiological studies suggesting a potential beneficial anti-inflammatory intervention [2], [3]–[4]. The initial stage of inflammation is associated with Aβ accumulation, microglia, astrocyte as well as complement system activation and increased production of proinflammatory cytokines [5]. Numerous investigations suggest that it is not only the central nervous system cells (microglia, astrocytes) that can be blamed for inflammatory response in AD, but also cells from the periphery, notably including the T lymphocytes (e.g. [6]–[8]).

Some of the recent investigations prove that T cells are able to enter the brain [9], despite a well-grounded knowledge that brain is an organ protected from systemic immune response. One of the hypotheses states that because of either a blood-brain barrier (BBB) damage or in response to signals originating from the cells being part of the inflammatory response in the brain (both of which are typical for AD pathology) lymphocytes infiltrate and accumulate in the places of Aβ localization in brain tissue.

Despite previous reports that T cells are able to enter the brain tissue, it is also possible that T cells exert their effect without entering the CNS. This action can be performed through T cell-secreted proinflammatory cytokines and their influence on microglia and astrocytes, or possibly through T cells promoting activation of monocytes and/or dendritic cells and consequent proinflammatory cytokines secretion. Both of these mechanisms can hypothetically participate in brain inflammation and gliosis once these proinflammatory cytokines cross BBB. During the initial stages of AD the immune system activity is directed towards elimination of β-amyloid, which results in anti-Aβ antibodies' production and inflammatory response. Despite the well-documented knowledge about inflammatory processes provoked by Aβ and immune dysregulation in AD, a number of questions remain unanswered concerning the activation state of peripheral T-cells, subsets of T cells which become activated and the timing of these events in the course of AD. We have shown before that the proportion of naïve to activated terminal memory T cells differs significantly in mild AD patients [10].

One of the key questions is whether the Aβ itself is able to trigger a specific, significant proliferative response of these cells which can contribute to the pathogenesis of AD. A number of investigations have proven positively that Aβ can play the role of antigen for T cells either in AD patients or in healthy people. Nevertheless, the question if β-amyloid can serve as an antigen for T cells remains unanswered, as some investigations report that Aβ1–40 does not trigger any proliferative reaction of T cells [11], and others showed that only T cells of healthy people, including old and young, are able to react to Aβ, whereas those from AD patients do not exhibit such action [12]. In the recent years, it was shown with the use of specially designed experiments for detection of autoreactive T lymphocytes using different Aβ peptides for stimulation (including Aβ1–42), that T lymphocytes reactive against β-amyloid circulate in the peripheral blood and their number is increasing with age [13]. Therefore previous studies seem highly inconsistent and haven't provided definite answer to the question of Aβ antigenicity in AD patients and healthy elderly.

The verification of hypothesis about possible stimulation of T cells by Aβ is especially vital in the context of previous, as it appeared not entirely successful, attempts to construct an efficient vaccination directed against β-amyloids. The latter prompted our hypothesis that, apart from it being an antigen triggering the usual response, Aβ may also exert more general modulatory effect on the immune system. In order to prove or disprove this hypothesis, we investigated here the population of peripheral blood CD4^+^CD28^+^ cells in order to check its proliferative response to three different Aβs (1–42, 1–40, and 25–35) simultaneously checking *ex vivo* their activation phenotype and potential to produce Th1 and Th2 cytokines in response to β-amyloid.

## Materials and Methods

### Patients

Samples of peripheral venous blood were obtained from 12 AD patients aged 52–96 (average 74±10) years and 15 age- and sex-matched controls (average age 67±7, range 58–78 years) who took part in the study.

All patients underwent detailed physical, neurological and psychiatric examination. Tests of cognitive functions and the evaluation of general functioning were done. Each of patients had a routine MRI or SPECT examination performed. Only patients meeting ICD-10 criteria of AD and criteria of probable AD according to revised research NINCDS-ADRDA criteria [14] were included in the study. Patients were subjected to longer clinical observations, which increased clinical diagnostic accuracy. Based on MMSE results there were 7 patients with moderate (MMSE = 14–22) and 5 with severe (MMSE<13) intensity of symptoms. Two of the patients (2/12) were treated with cholinesterase inhibitors (donepezil) at the time of study and for at least three months prior, while 10/12 were not treated with the drugs ever. Neither of the patients or controls had exhibited features of acute inflammatory process, including elevated ESR, CRP, or neutrophil count.

Healthy people above the age of 65 years conformed to the SENIEUR Protocol criteria [15]. All healthy controls underwent the Mini-Mental State Examination (MMSE) test with the score from 28–30 [16]. The exclusion criteria for healthy people included autoimmune diseases, neoplasms and acute or chronic inflammation. None of the healthy volunteers were taking medication that would influence the immune system. All participants were informed about the purpose of the tests and gave their written informed consent; the project was approved by the Local Committee for Biomedical Research Ethics at the Medical University of Gdańsk.

### Detection of T cell phenotype and activation status by surface marker staining

Ex vivo phenotype assessment was performed by staining one hundred microliters of peripheral blood for 30 minutes at room temperature with combinations of fluorescent FITC-conjugated anti-CD3, phycoerythrin (PE)-Cy5-conjugated anti-CD4 (both from DAKO Cytomation, Denmark), PE-conjugated CD28, CD25, CD69 and HLA-DR (all from Becton Dickinson Biosciences, USA). Proportions of the CD4^lo^CD25^high^(FoxP3^high^) regulatory T cells (Tregs) were estimated according to [17]. Staining with appropriate, irrelevant, fluorochrome-conjugated mouse antibodies of the same Ig class (DAKO and BD, as appropriate) was used as negative (isotype) control. Prior to analysis, the red blood cells were lysed in erythrocyte lysis buffer (BD Biosciences, USA) buffer according to manufacturer's protocol. The flow cytometry analysis was performed using FACScan flow cytometer (BD Biosciences, USA). All of the obtained flow cytometry data was analyzed using Win MDI 2.9 software (J. Trotter, The Scripps Institute, La Jolla, USA).

### Assessment of proliferative capabilities of the CD4^+^28^+^ lymphocytes using the DCT technique

PBMC were isolated by flotation over Histopaque™ (Sigma-Aldrich) and washed twice with RPMI-1640 medium (Sigma Aldrich, USA), containing 100 U/ml penicillin and 100 µg/ml streptomycin (PS) (Sigma Aldrich, USA). Cells were cultured in complete culture medium consisting of RPMI-1640 supplemented with 2 mM L-glutamine, 10% heat-inactivated fetal calf serum (FCS, Sigma Aldrich, USA), and the PS antibiotics.

For comparison of proliferative dynamic of treated and untreated lymphocytes the dividing cell tracking (DCT) technique [18] was adopted. Briefly, six to twelve million separated PBMC were stained with 2 µM CFSE (carboxyfluorescein diacetate succinimidyl ester) for 15 min at 37°C and subsequently washed twice with RPMI/PS mixture. Next, samples of 1 million of CFSE-stained PBMC were cultured in complete medium, at 37°C and 5% CO_2_, and in the presence of either 0.2 or 0.5 µM soluble amyloid peptides Aβ 1–42, Aβ 1–40 or a derived neurotoxic decapeptide Aβ 25–35 (Sigma Aldrich, USA). These concentrations were chosen based on the reported concentrations of free Aβ in the physiological fluids and on reported Aβ concentrations affecting cytokine secretion by human T cells *in vitro* [19], [20]. Similar, CFSE-loaded PBMC cultures were stimulated with immobilized anti-CD3 antibody (125 ng/ml, Becton Dickinson, USA), alone or in combination with previously listed Aβ peptides at 0.2 µM. In a pilot experiment we have found that neither of the two soluble Aβ concentrations used produced significant increase in PBMC apoptosis. Unstimulated PBMC cultured in complete medium were treated as negative control. After 72 and 120 hours the cells were gathered from the wells, counted, washed with PBS buffer, stained against CD4 and CD28 antigens with appropriate fluorochrome-conjugated antibodies listed above and FACS-analyzed. In the analysis of proliferative response we concentrated on the CD4^+^CD28^+^ population, which was appropriately gated during analysis. Analysis of proliferative dynamics of Aβ-treated and untreated CD4^+^CD28^+^ lymphocytes included the estimation of actual average number of divisions performed per a dividing lymphocyte, cell cycle timing and the G0→G1 transition time [18].

### Assessement of influence of the Aβ peptides on Th1/Th2 cytokine production *in vitro*

After 72 and 120 hours of PBMC stimulation with either anti-CD3 alone or in combination with Aβ peptides, culture supernatants were collected and frozen at −80°C for subsequent analysis. To assess the production of Th1/Th2 cytokines (IL-2, IL-4, IL-6, IL-10, TNF-α and IFN-γ) by the overall population of cultured PBMCs, the Cytometric Bead Array (CBA™, Becton Dickinson, USA) was used. Lyophilized cytokine proteins included in the kit were used to prepare standard curves. 3000 events were acquired for each sample and later analyzed with the use of Becton Dickinson (BD) cytometric bead array (CBA) software.

### Statistical analysis

Data were analyzed using a non-parametric test for unpaired (Mann-Whitney U test) or paired (Wilcoxon signed rank test) data. All statistical analyses were performed with the StatSoft, Inc. (2008) Statistica™ data analysis software system version 8.0, under the Medical University of Gdańsk license agreement. Significance levels are indicated by * when p<0.05, ** when p<0.005 and *** when P<0.0001 unless mentioned differently in the legends. K-means clustering was used to subgroup patients and healthy donors into groups of high and low responders considering the response towards Aβ1–42.

## Results

### β-amyloid peptides alone do not stimulate significant proliferation of CD4+ lymphocytes

**Figure 1:**
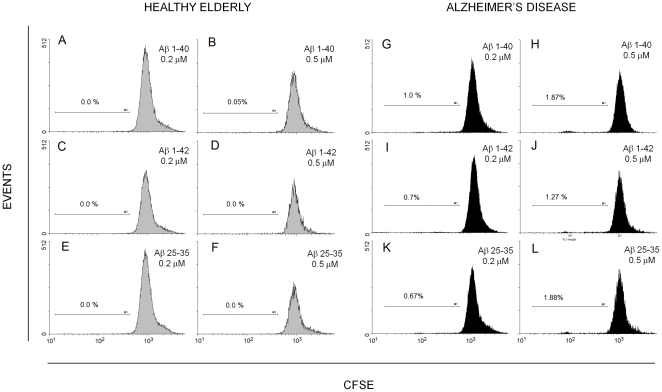
β-amyloid peptides do not stimulate divisions of healthy CD4^+^CD28^+^ lymphocytes and only weakly those of AD cells in vitro. CD4^+^CD28^+^ lymphocyte proliferation was measured with DCT technique as described in Materials and Methods, after stimulation for 120 hours with Aβ 1–40 (A,B,G,H), Aβ 1–42(C,D,I,J) or Aβ 25–35 (E,F,K,L) at 0.2 or 0.5 µM as indicated. Proportion of dividing cells is given in each histogram as the net percent of total CD4^+^CD28^+^ population, obtained by subtracting the observed proportion of proliferating (CFSE-diluting) lymphocytes in untreated sample from the proportions observed for relevant samples treated with the Aβs. Results of an experiment involving paired material from a healthy, matched control (left panel, gray histograms A–F) and from an AD patient (right panel, black histograms G–L), representative of 5 experiments giving similar picture, are shown.

When challenged *in vitro* with soluble β-amyloid peptides (Aβ1–42, Aβ 1–40, Aβ 25–35) only, the gross majority (on average, above 97%) of CFSE-loaded CD4^+^CD28^+^ lymphocytes of either healthy elderly or AD patients did not divide over 120 hours (Fig. 1). However, a closer scrutiny of the results had shown that, while Aβ peptides did not induce division of any CD4^+^CD28^+^ cells of healthy individuals (mean specific % of proliferating cells 0.0±0.0; Fig. 1, left panel), in the case of patient-derived lymphocytes some minor (usually below or around 1%), presumably specific (antigen-driven) proliferation could be seen in Aβ-challenged samples, especially if amyloids were used at 0.5 µM (mean specific % of proliferating cells 0.96±0.32; Fig. 1, right panel). Despite relatively low number of cases (n = 5) the difference in the effect of Aβs on healthy and AD lymphocytes was statistically significant (p = 0.017), presumably due to virtual lack of any effect of β-amyloid on the proliferation of healthy CD4^+^CD28^+^ cells.

### β-amyloid peptides significantly modulate proliferation of anti-CD3-stimulated lymphocytes

**Figure 2:**
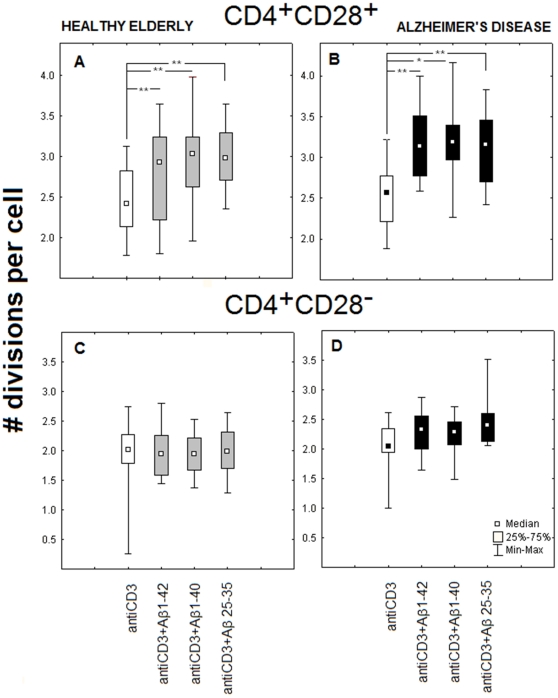
Proliferative dynamics of CD4^+^CD28^+^, but not of CD4^+^CD28^−^ lymphocytes is modulated by β-amyloids. Number of divisions per (dividing) cell for both healthy elderly (A) and AD (B) CD4^+^CD28^+^ cells significantly increased, while that of CD4^+^CD28^−^ lymphocytes (C, D) did not change after stimulation by anti-CD3 in combination with each of the three β-amyloid peptides at 0.2 µM. Cells were cultured as PBMC, and CD4^+^CD28^+^ lymphocytes were gated for DCT analysis based on appropriate phenotype staining as in Materials and Methods. Data are presented as medians, 25^th^ and 75^th^ percentiles and minimal/maximal values obtained for cells stimulated with immobilized anti-CD3 only (white boxes) or with added Aβ 1–42, Aβ 1–40, Aβ 25–35 (gray boxes for healthy and black ones for AD) as indicated. Statistically significant differences are marked with * (P = <0,05) or ** (P = <0,01).

**Figure 3:**
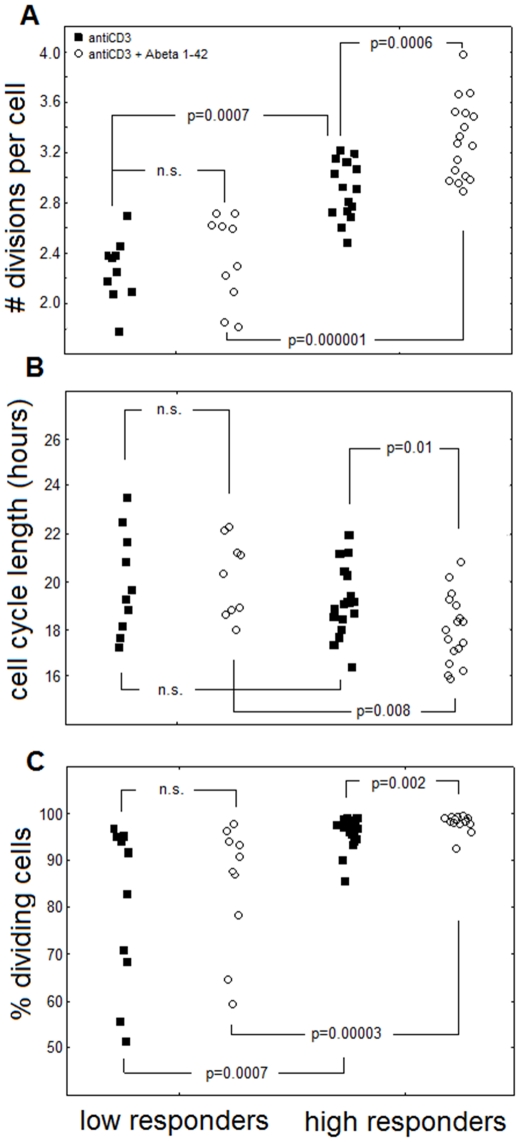
Healthy and AD individuals can be clustered according to high or low level of response to modulatory action of β-amyloids. Comparison of proliferative dynamic parameters of CD4^+^CD28^+^ cells from individuals clustered according to high (high responders) and low response to Aβ 1–42 (low responders) Cluster analysis for combined groups of AD patients and healthy elderly according to enhancement of proliferative response of CD4^+^CD28^+^ cells to anti-CD3+Aβ 1–42 stimulation was performed as in Materials and Methods. Highly significant differences between “high” and “low” responders can be observed regarding the numbers of divisions per cell (A), cell cycle length (B) and the percentages of dividing cells (C).Relevant p values are shown. Not significant differences are marked n.s.

PBMC of AD patients and healthy elderly, previously loaded with CFSE, were treated with immobilized anti-CD3 antibody alone or in combination with one of the Aβs at 0.2 µM. The DCT assay results clearly show that β-amyloid peptides are able to enhance the proliferative response of CD4^+^CD28^+^ lymphocytes to anti-CD3, significantly increasing the effectiveness of stimulation by elevating the number of divisions per a dividing CD4^+^CD28^+^ lymphocyte (Fig. 2 A, B). Comparable results were obtained for AD patients and healthy elderly individuals, and for all three of the β-amyloids used: 1–42, 1–40 and 25–35, all of which were enhancing the proliferative response of CD4^+^CD28^+^ lymphocytes to anti-CD3 antibody. No such effect could be seen if the CD4^+^CD28^−^ lymphocytes from the same samples were analyzed (Fig. 3 C, D). Similar results were obtained when 0.5 µM concentrations of amyloids were used (not shown).

Even though we thus demonstrated the modulatory effect of every tested β-amyloid for CD4^+^CD28^+^ cells, we found no significant differences between AD patients and healthy elderly volunteers concerning the enhancement of cell cycle parameters neither in the numbers of divisions per cell nor in other dynamic features of cell cycle available in the DCT method [18], including proliferation index, number of cell precursors, the G0→G1 transition time or the percentage of proliferating cells (not shown).

The above might have resulted from our observation that in both investigated groups (AD vs. healthy elderly) the observed effects of Aβ were grossly individually variable and suggested intra-group differentiation. Aiming to find possible mechanism(s) explaining these data we performed cluster analysis and found two distinct groups, significantly differing in the assessed level of response (measured as number of divisions per a dividing cell) to anti-CD3 in combination with either 0.2 or 0.5 µM Aβ 1–42 after 120 hours of stimulation.

### Subject clustering based on different proliferative responses to modulation by Aβ reveals subdivision of both patients and healthy individuals concerning levels of T cell activation and cytokine secretion

Accordingly, we clustered the AD patients and healthy elderly into groups of high and low responders (Fig. 3). While the numbers of divisions per a CD4^+^CD28^+^ lymphocyte measured in the Aβ1–42 - treated PBMC from high responders differed significantly from both low responders and from controls stimulated with anti-CD3 only (signifying a strong modulatory effect of the Aβ on the lymphocytes of these individuals only), the number of divisions of ‘low responder’ lymphocytes observed when Aβ 1–42 was present or absent in the cultures did not differ (Fig. 3 A). Similarly, we have seen a significant shortening of the cell cycle length only if ‘high responder’ lymphocytes challenged with the Aβ were compared with those stimulated with anti-CD3 only or with ‘low responder’ cells stimulated with both Aβ and anti-CD3 (Fig. 3 B).

Finally, when the productivity of stimulation was assessed by the proportion of CD4^+^CD28^+^ cells that underwent at least one division in the total population of PBMC stimulated over 120 hours, it was significantly higher for ‘high responder’ than for ‘low responder’ lymphocytes; between 90 and a 100% of the former divided, while in a half of the latter the relevant proportion was below 90% and in some was as low as 50–60% only (Fig. 3C). Thus, significant, multi-faceted, response-enhancing effect of the Aβ could be observed only for high responders. It is worth noting here that compared to low responders, the high responders' T cells respond significantly more vigorously also to anti-CD3 only.

The group of high responders selected on the basis of k-means clustering included 9 AD patients and 8 healthy elderly whereas the group of low responders consisted of 3 AD patients and 7 healthy donors. Interestingly, despite apparently higher proportion of low responders among the healthy, the healthy and AD groups did not differ significantly in these proportions (Fisher's exact test, p = 0.69). Also, the average division numbers did not differ between AD and healthy individuals categorized as high or low responders (not shown).

It is well known that ageing results in modification of many properties of the immune cells, including the proportions of CD4^+^CD28^+^ lymphocytes. Thus, theoretically, observed differences between the ‘high’ and ‘low responders’ could be the result of nonrandom distribution of age and/or proportion of the CD4^+^CD28^+^ cells. As shown in the Materials and Methods section, our subjects' age range was between 52 and 96 years. Still, the high responder and low responder groups did not differ significantly regarding their respective average ages. Also, we did not see significant correlation between subject ages and any of the parameters discriminating the high and low responders. Interestingly, high responders exhibited statistically significant, higher proportion of the CD4^+^CD28^−^ lymphocytes (thus, reciprocally, lower proportion of the CD4^+^CD28^+^ cells) than low responders (medians 6.44% and 0.61% respectively; Mann-Whitney U test p = 0.046).

**Figure 4:**
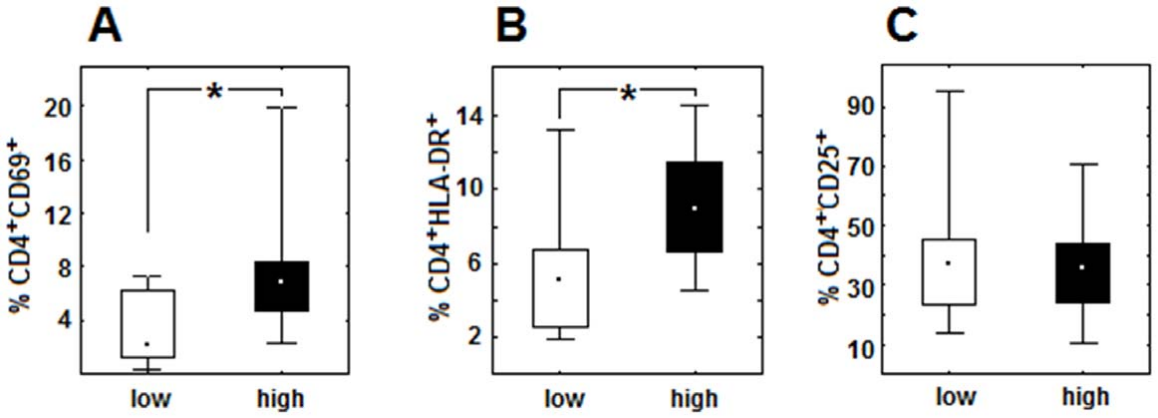
Activation status of the CD4^+^ lymphocytes of individuals exhibiting high or low response to Aβ is different. Percentages of CD4^+^ cells bearing activation markers: CD4^+^CD69^+^ (A) and CD4^+^HLA-DR^+^ (B) ex vivo are significantly elevated in the group of high responders (high) in comparison with low responders (low). No differences exist concerning CD4^+^CD25^+^ percentage between two investigated groups (C). Data are presented as medians, 25^th^ and 75^th^ percentiles and minimal/maximal values. Statistically significant differences are marked with * (P = <0,05).

Thus, in order to further characterize the high and low responders, we have analyzed the *ex vivo* phenotypes of their CD4^+^CD28^+^ cells and found that they significantly differed concerning the expression of activation antigens, including early expressed CD69 and, especially, the mid-to-late expressed HLA-DR, percentages of both being significantly higher in the group of high responders (Fig. 4 A and B). We found no differences between these two groups concerning another investigated activation antigen - CD25 (Fig. 4 C). It is noteworthy that the distribution of results obtained for AD and healthy individuals was random and homogenous within the ‘high’ and ‘low responder’ groups and thus it did not allow for their further subdivision according to ‘healthy’ or ‘AD’ status.

**Figure 5:**
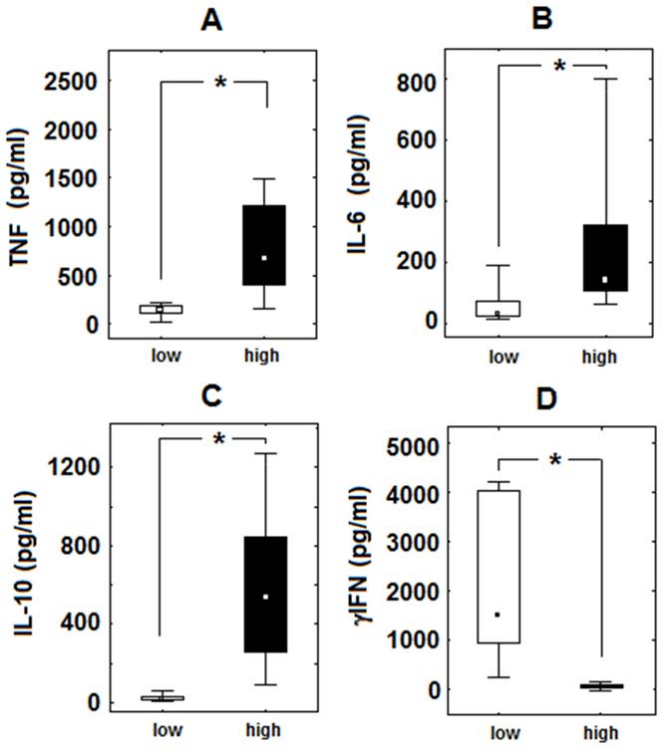
Cytokine production in vitro is different for cells of individuals exhibiting high or low response to Aβ. Modified levels of TNF, IL-10, IL-6 and IFN-γ produced by PBMC population in vitro after stimulation with anti-CD3 and β-amyloid 1–42 in the group of high vs. low responders. Data are presented as medians, 25^th^ and 75^th^ percentiles and minimal/maximal values as indicated. Statistically significant differences are marked with * (P<0,01).

After revealing that the CD4^+^CD28^+^ lymphocytes of high responders exhibits significantly elevated levels of activation-related antigens we checked wheather they differed also in Th1/Th2 cytokine production in response to anti-CD3 plus Aβ 1–42. We found that cultured PBMC of high responders produced significantly higher levels of TNF-α (Fig. 5 A), IL-6 (Fig. 5 B) and IL-10 (Fig. 5 C). On the other hand, they made a significantly reduced level of IFNγ (Fig. 5 D). The cells from high responders, especially those triggered with both anti-CD3 and *β*-amyloid peptide, showed also a tendency to make higher amounts of IL-2 and IL-4 compared to low responders; however, the differences had not reached statistical significance in either case (not shown).

Differing functional (proliferative and secretory) properties of the CD4^+^CD28^+^ lymphocytes of high and low responders could also be the result of different proportion of regulatory T cells (Tregs) in the two groups. However, the proportions of the CD4^lo^CD25^high^ Tregs did not differ significantly between the high responder and low responder groups (not shown).

Summary of major differences between high and low responders is shown in the Table 1.

**Table 1:** Major features of high responders and their CD4^+^ lymphocytes as compared to low responders.

	CLINICAL FEATURES		RESTING PHENOTYPE OF CD4^+^ LYMPHOCYTES				RESPONSE TO ANTI-CD3 STIMULATION					
							Proliferation		Cytokine secretion			
	Age	MMSE	CD28^−^	CD25^+^	CD69^+^	HLA-DR^+^	−Aβ	+Aβ	IFNγ	TNF	IL-6	IL-10
High responders vs. low responders	N.S.	↓	↑	N.S.	↑	↑	↑	↑↑	↓	↑	↑	↑

Value observed for high responders is shown as significantly higher (↑,↑↑) or lower (↓) than observed for low responders.

N.S. – lack of statistically significant difference between high and low responders.

## Discussion

Although the disturbances of the immune system and the presence of systemic inflammation associated to the Alzheimer's disease have been widely studied and thoroughly documented in numerous papers, the understanding of their role in its pathogenesis is far from complete. The immune system engagement in AD is especially interesting concerning continuing attempts towards constructing a vaccine aiming to eliminate β-amyloid, which appeared successful in mice but had dramatic effects in the clinical studies in humans. In AD mouse models immunization with aggregated β-amyloid 1–42 peptide reduced cerebral β-amyloid deposition, neuritic dystrophy and gliosis in amyloid precursor transgenic (APP-tg) mice [21]–[23] and also improved cognition [24],[25]. Clinical study in AD patients using aggregated Aβ1–42 in combination with QS21 adjuvant was halted because of signs of meningoencephalitis in 6% of the patients [26]. Nevertheless, those patients whose response to vaccination was positive and resulted in anti-Aβ antibodies production presented slower cognitive decline [27], [28]. Further investigations revealed that two patients with encephalitis had T-cells infiltrates in the brains which suggest T cell-mediated immune response as a reason for the adverse events [29], [30].

There are hypotheses suggesting persistent stimulation of immune system by Aβ peptides leading to T cell response as well as to the release of inflammatory mediators and changes in the activation levels of T cells. In our experimental model we found that 0.2 or 0.5 µM Aβ peptides characteristic for AD neuropathology (Aβ 1–42 and 1–40) as well as the derived neurotoxic decapeptide Aβ 25–35, are (even if not previously aggregated) by themselves able to trigger a presumably specific proliferative response of only a small proportion (around 1%) of CD4^+^CD28^+^ lymphocytes over up to 120 hours of contact in vitro. This relatively scarce (but still significant) response was seen only for patients' cells and not for those from healthy controls (for whom the response was virtually null). We have employed the DCT method based on CFSE staining, enabling to trace specific proliferation of phenotypically defined cells, while not requiring separation of cells of interesting phenotype from PBMC mixture neither prior nor after stimulation [18]. On the other hand, previous investigations based on ^3^H-thymidine incorporation as a method of estimating cells proliferation, proved to be highly incoherent. Some of them postulate hyper-responsiveness of T cells from healthy elderly individuals and AD patients in comparison with middle aged healthy donors [13], while other claim that there are no significant differences in the ability of T cells proliferative response to Aβ 1–42 in the abovementioned groups [31] or that T cells of healthy individuals respond to Aβ peptides while those from AD patients do not [32]. Finally, the authors of some studies show that Aβ 1–40 and 1–42 do not provoke T cell proliferative response [11]. A recent paper by Zota et al. associates the ability to respond specifically to Aβ with the expression of HLA-DRB1*1501 allele [33]. Although we did not type the HLA-DR of our subjects, probability that all of the AD patients were HLA-DRB1*1501 positive, and the controls did not exhibit this haplotype is extremely low; thus, we suggest that HLA makeup had no influence on our results. Our observations employing a technique making possible to distinguish (virtually “see” in the FACS plots) and quantify single dividing lymphocytes seem to support the ability of CD4^+^CD28^+^ cells of AD patients to specifically proliferatively respond to Aβ peptide, but at a level not exceeding that seen for other antigens. This is clearly enough to elicit also the specific antibody response as seen in Aβ vaccination tests [27], [28], even if the increase in IL-4 production by Aβ-challenged lymphocytes that we were able to show was not significant. We confirmed the proliferative potential of the investigated AD cells using immobilized anti-CD3 antibody stimulation and finding no significant difference between the proliferation dynamics of CD4^+^CD28^+^ lymphocytes of AD and healthy elderly individuals. We believe that in our experimental model employing total PBMC, the antigenic properties of β-amyloid peptides could be exhibited both through monocyte/macrophages as antigen presenting cells or directly as it is possible in case of Aβ 25–35 decapeptide, which is apparently not presented by the APCs [34].

The level of reaction elicited by soluble Aβ peptides admixed with anti-CD3-stimulated cultures, compared to stimulation with anti-CD3 only, seemed to be very variable between investigated subjects. Based on k-means clustering of proliferation parameters, we distinguished a group of “high” and “low” responders in both investigated groups and found that only the high responders' cells exhibited significantly activated CD4^+^CD69^+^ and CD4^+^HLA-DR^+^ phenotypes. The difference between high and low responders was not related to age of individuals included in both groups, or to the proportion of T regulatory (Treg) lymphocytes. Comparison of the ability of lymphocytes from high and low responders to produce the Th1, Th2, and proinflammatory cytokines *in vitro* revealed that the high responder cells produced significantly more TNF-α, IL-6 and IL-10, and significantly less IFNγ than low responders. Lower production of IFNγ by high responders' cells seems also to fit well with the notion of the role of this cytokine in stimulating the clearing of Aβ deposits [35], [36]; thus, high responders would be at higher risk of amyloid plaque accumulation and, possibly, faster progression of the disease. Such a possible relation requires further study. Accordingly, we have compared the results of MMSE test for the AD patients belonging to high and low responders and found a trend towards lower values in the high responders (mean MMSE values being 21.8±9.7 for high and 25.4±7.4 for low responders), which seems to corroborate our suggestion of more active disease in the high responders.

TNF-α levels were earlier found to be increased not only in the brain but also in the cerebrospinal fluid and in the blood plasma of patients suffering from AD [37]–[38], [39]. Also, a recent study demonstrated an increased production of IL-6 and IL-10 in mild cognitive impairment suggesting that immune activation is an early phenomenon that precedes AD [40]. Contrarily, Schott et al in their investigations in activated blood cell cultures report significant decrease of pro- as well as anti-inflammatory cytokines in AD group and suggests the general decline of immune responsiveness in AD [41]. Despite seeing significant differences between the high responder and low responder cells in the proportions of activated lymphocytes (CD4^+^CD69^+^, CD4^+^HLA-DR^+^) and of CD4^+^CD28^−^ cells, we cannot attribute decreased production of IFNγ and increased secretion of other proinflammatory cytokines by the high responder lymphocytes to these phenotypic shifts. In particular, observed tenfold increase of the proportion of CD4^+^CD28^−^ lymphocytes in high responders should rather lead to an increase in IFNγ production, as these cells are known to manufacture significant amounts of this cytokine (e.g. [42], [43] ). Thus, we hypothesize that a regulatory phenomenon occurring in the major, CD4^+^CD28^+^ population, possibly due to different response of high- and low responders to Aβ peptides in vivo, is responsible for unusual cytokine secretion pattern.

Observed pattern of cytokine synthesis suggests general pro-inflammatory and ‘pro-AD’ shift in the reactivity of susceptible (high responder) cells challenged with Aβ which may lay the ground to otherwise unpredictably higher inflammatory response seen in some individuals challenged with Aβ vaccine. Apparently, concomitant increase in the level of anti-inflammatory IL-10, reported also by other studies [44], is not sufficient to alleviate the inflammatory process happening in the CNS. In our opinion, successful vaccination in mice could be observed as the experimental animals were reared in a relatively pathogen-free environment of ‘specific pathogen free’ animal house; thus, their immune systems remained at low levels of activity possibly preventing the generalized modulatory effects of Aβ. Contrarily, human immune system in a typical environment is constantly (sub-clinically) coping with multiple environmental pathogens, making it generally more activated. Thus, cells stimulated *in vitro* with anti-CD3 would be a better model of actual condition of the typical human immune system, compared to the murine one.

**Figure 6:**
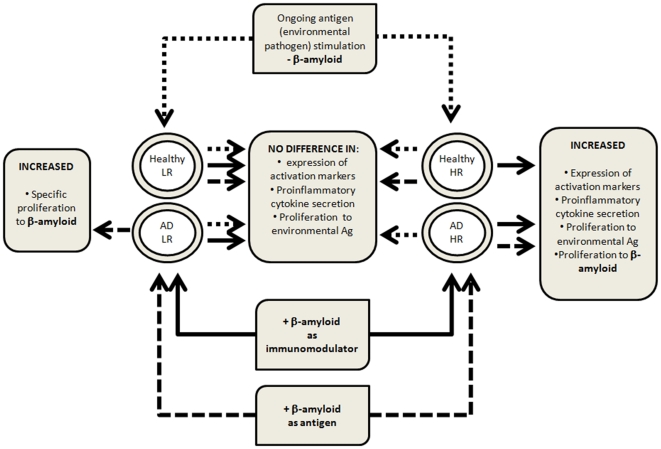
Proposed model explaining the difference in reactivity to Aβ during *in vivo* and *in vitro* stimulation between the CD4^+^CD28^+^ lymphocytes of high (HR) and low responders (LR). Arrows connect the type of responder (high vs. low) with expected response to stimulation. Dotted arrows: environmental Ag stimulation and response; dashed arrows: Aβ-specific (antigenic) stimulation and response; solid arrows: Aβ - immunomodulatory response.

Our proposed model based on different reactivity of high and low responders' lymphocytes to Aβ is shown in Fig. 6. It assumes that in addition to proliferative reaction to the presence of (at least initially soluble) Aβ by the T cells (of AD patients only) specifically expressing Aβ-specific TCR, the amyloid is recognized (and reacted to) differently by the CD4^+^CD28^+^ but not CD4^+^CD28^−^ cells of high and low responders. It is worth mentioning here that high and low responders differed in the proportion of their CD4^+^CD28^−^ lymphocytes, which was significantly lower in the latter. In our opinion, different proportion of these cells, known to proliferate less vigorously than CD4^+^CD28^+^ counterparts and to exert rather a suppressive and cytotoxic activity, does not participate in the establishment of high or low responder phenotype. Thus, T lymphocytes of all AD patients would respond to Aβ specifically, while those of high responders among AD patients would respond also nonspecifically; cells of healthy elderly controls would only respond nonspecifically, if an individual belongs to the ‘high responders’ group. Hypothetical mechanism of this non-specific immunomodulation can be associated with direct contact of activated CD4^+^CD28^+^ cells with soluble Aβ through nonspecific receptors. Their nonspecificity postulated here excludes the possibility of any role of various HLA haplotypes in the phenomenon, including HLA-DRB1*1501 shown to be associated with specific response to Aβ [33].

It is worth mentioning here that, although it is the aggregated Aβ which is found in the brain amyloid plaques and causative for neurotoxicity, it is the soluble (mono- to oligomeric) form that predominates in the plasma, due to anti-aggregation action of albumins [45], [46]. Thus, soluble forms of Aβ would be predominantly in contact with the immune cells outside the brain, making them more likely to exert modulatory activities postulated and demonstrated in this work. In fact, most papers where Aβ was used to stimulate the immune cells in vitro utilized its initially soluble form. Although it cannot be excluded that some Aβ aggregation could happen during the cell culture in both this and other works, it should be relatively minor due to presence of albumin from bovine serum constituting a component of the culture medium. Eventual aggregates, if formed, should be internalized by monocyte/macrophages present among the PBMC in culture, then properly elaborated and presented in the context of HLA; this process should be responsible for the specific response we recorded (admittedly minor and not different among AD and healthy individuals).

Potential soluble Aβ receptors can be for example the RAGEs (receptors for advanced glycosylation end products) present on CD4^+^ T cell surface and known to bind various molecules including the Aβ; ligation of RAGE results in cells activation and inflammatory response [47], [48]. One cannot exclude also the role of TLR4 [49] and possibly oxLDL-R (CD36) [50] in the phenomenon. TLR-4 receptors are expressed on human CD4+ lymphocytes [51], so their potentially modulatory effect upon ligation by Aβ may even be direct.

An interesting alternate hypothesis, possibly explaining different behavior of Aβ – challenged lymphocytes from high- and low-responders, arises from recent reports on soluble Aβ being bound by surface-expressed prion precursor protein PrP^c^ [52], [53]. It is already known for more than a decade, that PrP^c^ is expressed on the surface of human peripheral blood lymphocytes (including CD4^+^ cells) and that its expression increases in stimulated lymphocytes after that of e.g. CD69 and CD25, making the PrP^c^ a late activation marker, possibly assuring a fine-tuning of the immune response by modulating calcium signals during the activation of cells when triggered with appropriate ligand [54], [55]. The latter agrees well with the findings stating increased initial intracellular calcium levels in T cells from patients with AD set against age-matched controls [56]. Thus, individuals whose CD4^+^ lymphocytes would strongly (for high responders) or weakly (for low-responders) modulate their proliferative and cytokine-producing functions when exposed to Aβ, should differ in the expression level of the PrP^c^ or in the levels of associated signaling. A likelihood of this hypothesis (admittedly, requiring further studies to prove or disprove it) is augmented by finding of ageing-associated increase in the level of PrP^c^ expression on human lymphocytes [57] and of its relatively higher expression on memory as compared to naïve cells [54]. We have recently shown significantly increased proportions of memory CD4^+^ lymphocytes circulating in the peripheral blood of AD patients [10]. As (despite huge range of subjects age in both AD and healthy groups) we did not see much relation between individual age and high- or low responder behavior of the T cells, the lymphocytes from high and low responders could differ in the levels of either of the abovementioned molecules (or any combination of these) due to e.g. individual genetic makeup.

Expression levels of CD69 of both T-cells and B cells correlated inversely with the results of Mini-mental Examination Scale tests in AD subjects, suggesting a direct relation between immune system activation and the clinical status of patients [58]. Our results, especially those showing increased activation markers' expression on the CD4^+^ cells of high responders from both healthy and AD groups, suggest that susceptibility towards activation induced by anti-CD3 combined with β-amyloid peptides is associated with initial activation status of CD4^+^ cells and not necessarily with presence of Alzheimer disease itself.

Numerous studies have shown that plasma (soluble to oligomeric) β-amyloid forms are present in AD subjects as well as the healthy individuals, with the significantly lowered plasma β-amyloid 1–42 in demented patients (e.g. [59]). This shows that soluble Aβ is accessible for circulating T cells in AD patients as well as healthy elderly. Not surprisingly at least some individuals in both of these groups possess CD4^+^CD28^+^ cells reacting by an enhanced proliferative response through continuous contact with Aβ as an endogenous, chronic stimulus. Interestingly, this up-modulation is limited to the ‘high responder’ subgroup that we propose and irrespective of the AD status.

In conclusion, our results seem to show that individuals differ in the susceptibility of their CD4^+^CD28^+^ lymphocytes to the modulatory effect of Aβ in their response to stimulation. The proportion of those who are susceptible (our ‘high responders’ does not vary significantly between the AD and clinically healthy cohort. Whether the high responder status may be explanatory for AD pathogenesis and progression or have a predictive value in prediction or early diagnosis of AD remains to be shown.
